# SiamPKHT: Hyperspectral Siamese Tracking Based on Pyramid Shuffle Attention and Knowledge Distillation

**DOI:** 10.3390/s23239554

**Published:** 2023-12-01

**Authors:** Kun Qian, Shiqing Wang, Shoujin Zhang, Jianlu Shen

**Affiliations:** School of Artifical Intelligence and Computer Science, Jiangnan University, Wuxi 214122, China; shiqingwang@stu.jiangnan.edu.cn (S.W.); 6223110057@stu.jiangnan.edu.cn (S.Z.); jlshen@stu.jiangnan.edu.cn (J.S.)

**Keywords:** hyperspectral video, target tracking, SiamCAR network, pyramid shuffle attention, knowledge distillation

## Abstract

Hyperspectral images provide a wealth of spectral and spatial information, offering significant advantages for the purpose of tracking objects. However, Siamese trackers are unable to fully exploit spectral features due to the limited number of hyperspectral videos. The high-dimensional nature of hyperspectral images complicates the model training process. In order to address the aforementioned issues, this article proposes a hyperspectral object tracking (HOT) algorithm callled SiamPKHT, which leverages the SiamCAR model by incorporating pyramid shuffle attention (PSA) and knowledge distillation (KD). First, the PSA module employs pyramid convolutions to extract multiscale features. In addition, shuffle attention is adopted to capture relationships between different channels and spatial positions, thereby obtaining good features with a stronger classification performance. Second, KD is introduced under the guidance of a pre-trained RGB tracking model, which deals with the problem of overfitting in HOT. Experiments using HOT2022 data indicate that the designed SiamPKHT achieves better performance compared to the baseline method (SiamCAR) and other state-of-the-art HOT algorithms. It also achieves real-time requirements at 43 frames per second.

## 1. Introduction

**Figure 1:**
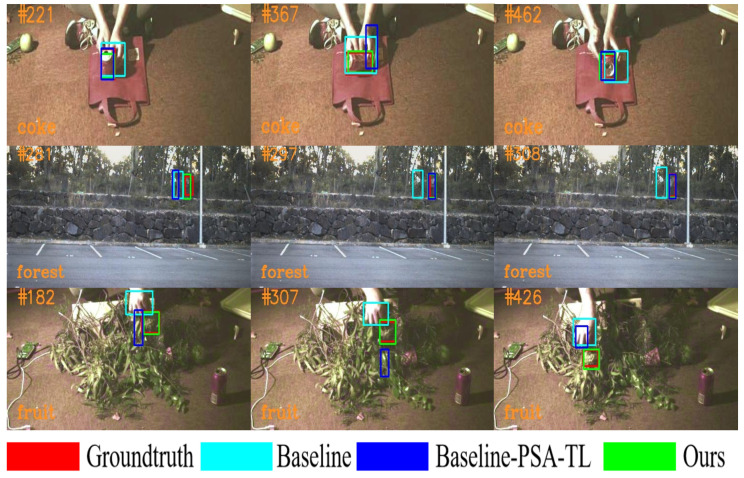
Our tracker compared to the baseline algorithm [6]. Our algorithm effectively addresses challenges, such as deformation, severe occlusion, and fast motion.

Single object tracking is a significant research direction in the field of computer vision [1,2,3,4], which aims to continuously track a specific object based on the information from the initial frame. Visual object tracking has made certain progress, yet it still encounters challenges, including target occlusion, appearance variations, fast motion, and scale changes. This is due to the fact that RGB-based trackers are limited to using only the red, green, and blue (RGB) color bands, which constrains their capability in feature representation. Hyperspectral imaging technology, on the other hand, captures the reflected or emitted spectra of objects at various wavelengths, offering more comprehensive spectral information [5] than RGB. This feature enables hyperspectral tracking to excel in distinguishing objects with subtle color differences and in scenarios where environmental lighting conditions change. Nowadays, it has become easier and more cost-effective to acquire hyperspectral videos (HSVs), thus providing opportunities for hyperspectral object tracking (HOT). As shown in Figure 1, an HOT algorithm that obtains material information is capable of identifying objects with similar appearances.

Early HOT algorithms were based on correlation filtering (CF). Uzkent et al. [7] introduced an HOT method based on deep kernelized correlation filters (DeepHKCF) that effectively tracks airborne targets with an adaptive multimodal hyperspectral sensor. However, the method leads to the reduction of spectral information when processing hyperspectral images (HSIs), thereby reducing the performance of the tracker. Qian et al. [8] utilized convolutional kernels for feature extraction by extracting a collection of patches around the object in each spectral band. However, this approach neglects the correlations that exist between different spectral bands. Xiong et al. [5] proposed a material-based hyperspectral tracker (MHT) that incorporates the spectral-spatial information of HSVs into a multidimensional oriented gradient histogram. This technique enables an accurate representation of the target by utilizing global material features, but the performance of the tracker needs to be improved in scenes of illumination variations. Zhang et al. [9] took advantage of multi-feature integration using spatial, spectral, and temporal information. Although the integrated feature is strong in discriminating the target from the background, parameter tuning requires a large amount of experimental optimization. Recently, Zhao et al. [10] proposed a method based on pixel-wise spectral matching reduction and deep cascading of spectral textural features, successfully mitigating the effects caused by illumination.

Compared to CF trackers, deep learning (DL)-based trackers demonstrate superior performance in terms of accuracy. Li et al. [11] introduced an HSV tracker based on the Band Attention-aware Ensemble Network (BAENet), which can obtain several groups of spectral bands. However, BAENet tends to lose the target in the case of scale variations. Wang et al. [12] combined band selection with an improved Siamese [3] network. Their genetic optimization model helped to obtain three bands according to the optimal joint entropy, and then transfer learning (TL) enhanced the antideformation capability referring to object tracking. Nevertheless, the generalizability of this method is not strong enough. Furthermore, Li et al. [13] proposed a Siamese network of Band Attention-based Grouping (SiamBAG), which combines all bands into many 3-channel images to exploit all spectral features, but it is time consuming. In addition, Li et al. [14] introduced a deep ensemble Network using Spectral Self-Expression (SEENet), with which the importance of each spectral band can be obtained. It is worth mentioning that a dynamic aggregation module is used to integrate the prediction results of each recomposed image, suppressing unreliable tracking referring to low-importance images.

Today, researchers address HOT by integrating deep features into CF-based trackers. The core of DL-based trackers is based on training large amounts of data, whose adequacy has a substantial effect on tracking performance. Most DL-based HOT methods use models developed on RGB datasets as backbone networks. However, it is not possible to directly apply models trained on visual images to HSVs due to the difference in the number of bands. Generally, two approaches are utilized to deal with this issue. One approach is to convert HSV to false-color images [13,15]. However, this method loses some spectral information. An alternative strategy tends to decompose the HSI into a series of images [14,16], which is accomplished by sequentially organizing the spectral band. However, the information becomes redundant as a result of its strong similarity to adjacent bands. Additionally, the high computational cost poses challenges in meeting real-time requirements. Due to the scarcity of hyperspectral data, fine-tuning for TL tends to compromise the original robustness of the model, rendering the tracker more susceptible to noise or other forms of interference.

**Figure 2:**
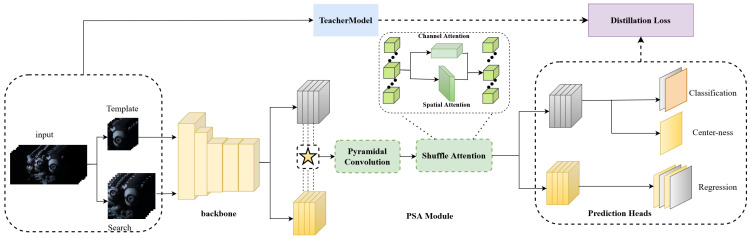
The framework of the proposed SiamPKHT algorithm. It consists of four components: feature extraction, a PSA module for similarity enhancement, prediction heads for target box prediction, and hyperspectral knowledge distillation (HKD).

From the above analysis, we introduce an HOT approach that combines pyramid shuffle attention (PSA) and knowledge distillation (KD) in a Siamese framework, namely SiamPKHT. The proposed framework is illustrated in Figure 2 and mainly includes four components: feature extraction, similarity enhancement, target box prediction, and hyperspectral knowledge distillation (HKD). In our previous work, as detailed in [17], we employed a genetic algorithm to identify three spectral bands with the highest joint information entropy. These bands were used to create false-color images, which we then input into the ResNet50 network [18]. This method effectively captures essential spectral data while minimizing redundant information in HSVs, thus speeding up the computations. Then, the initial similarity features were obtained by cross-correlating the template and search region features. Furthermore, a PSA module was applied to improve similarity features. The PSA module, by integrating multi-scale information regarding similarity features and capturing pixel-level pairwise relationships and channel dependencies, enabled the model to more effectively handle the complexity of hyperspectral data. This also enhanced the model’s generalization capability, allowing it to better cope with a diverse range of real-world application scenarios. Therefore, classification and regression maps were obtained to forecast the target state. Moreover, the proposed model incorporated KD to transfer hyperspectral information through TL. A teacher model, trained on a color image dataset [19], served as a guide in the training phase. Additionally, a distillation loss was designed to avoid overfitting problems from insufficient training samples. Compared to previous works [12,16], employing KD techniques aids the model in learning more profound levels of feature abstraction, significantly enhancing training efficiency. In summary, the proposed SiamPKHT tracker trained by this method effectively achieves better generalization.

**Figure 3:**
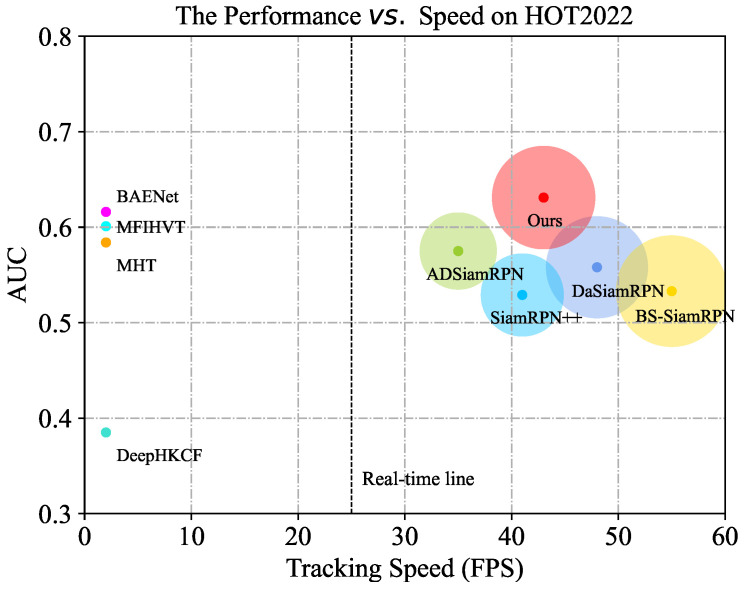
The performance comparison with several SOTA trackers on HOT2022. We visualize the area under the curve (AUC) relative to frames per second (FPS). The radii of the circles represent the speed of the trackers.

As shown in Figure 3, the proposed algorithm outperforms other state-of-the-art (SOTA) trackers significantly with respect to the area under the curve (AUC) [20] while meeting the requirements of real-time tracking. The key contributions of this paper are described below.

- The hyperspectral target tracking algorithm, which uses SiamCAR as the backbone, works at 43 FPS, meeting real-time requirements.
- A feature enhancement module based on pyramid shuffle attention is designed to increase the representation capability of similarity maps by establishing relationships between features and fusing multiscale information.
- To effectively alleviate the problem of overfitting, we design a hyperspectral knowledge distillation training approach that benefits from RGB datasets.
- Comprehensive experiments carried out with the HOT2022 benchmark demonstrate that PSA and HKD can improve the performance of the baseline method. Furthermore, the proposed tracker achieves exceptional performance in all HSVs.

## 2. Related Work

This section will review three main aspects relevant to our work: Siamese trackers, the mechanism of attention, and the distillation of knowledge.

### 2.1. Siamese Trackers

Recently, Siamese networks have been widely used in single-object tracking [3,21,22,23,24,25,26,27,28,29]. The primary idea behind the Siamese tracker is to track a target by comparing similarities between features from both the template and the search region. This method employs a Siamese network architecture with shared weights, where two identical networks jointly learn the representation of the target. Siamese Instance Search Tracker (SINT) [21] is a pioneering Siamese tracker that utilizes dual branches of an identical backbone network to produce feature maps. Fully convolutional Siamese (SiamFC) networks [22] are innovative Siamese trackers that first utilize the cross-correlation layer to merge information from two different branches. They employ template features as convolutional kernels to perform convolutional operations over the search area, obtaining similarity features between both parts. This similarity map is utilized for the prediction of the target position. The Siamese Region Proposal Network (SiamRPN) [3] introduces a region proposal network to achieve more precise tracking with high speed. However, the task faces the challenge that a distractor is similar in appearance to the object of interest. Zhu et al. [24] proposed Distractor-aware SiamRPN (DaSiamRPN), which utilizes data augmentation strategies to address the issue of imbalanced distribution referring to training data. The introduction of deep networks into the Siamese framework for feature extraction was pioneered by SiamRPN++ [23]. In addition, an innovative deep correlation layer proposed in SiamRPN++ can efficiently incorporate information from branches. These anchor-based methods have to spend much time searching for the appropriate anchor parameters.

To avoid these issues, anchor-free models have been adopted to directly forecast bounding boxes rather than relying on pre-established anchor boxes. Ocean et al. [30] proposed an anchor-free network with object awareness, which estimates the position of the target by forecasting the distance of pixels from the object box. This mechanism enables the learning of object-aware features, thus improving the tracking performance. Chen et al. [31] proposed a flexible Siamese Box Adaptive Network (SiamBAN) that allows for end-to-end offline training, thus avoiding parameter tuning referring to candidate boxes. Furthermore, SiamFC++ [32] adopts a privileged estimation quality assessment branch, which focuses on high-quality bounding boxes. SiamCAR [6] describes a supplementary centerness branch alongside the classification branch and helps to deal with the problem of predicted bounding boxes that are substandard. In order to reduce the time required for the anchor point parameter search, we have adopted SiamCAR as the backbone of our approach. Generally speaking, our strategy is applicable to all tracking methods based on Siamese networks, as Siamese networks share common characteristics, such as target feature extraction and similarity measurement. Further experimental validation is needed to confirm this.

### 2.2. The Attention Mechanism

The study of attention mechanisms can be traced back to early research on visual attention, which focuses on important objects in visual images. As DL gains popularity, attention mechanisms have been introduced into neural networks, providing the ability for models to dynamically focus attention. Recently, attention mechanisms have gained widespread applications and research interest in computer vision [33,34,35,36,37,38]. Specifically, Squeeze-and-Excitation Networks (SENet) [39] enhance representational capacity by integrating spatial and channel features in local receptive fields to extract informative features. The Convolutional Block Attention Module (CBAM) [40] effectively enhances intermediate features by sequentially deducing attention maps over both the spatial and the channel dimensions. The Residual Attentional Siamese Network (RASNet) [41] integrates spatial and channel attention to increase the capacity to discern features.

In the field of HOT, attention mechanisms have also found widespread applications in enhancing performance. For instance, SiamBGA [13] and BAENet [11] utilize band attention blocks to discern relationships among spectral bands, segmenting HSIs into multi-channel images for tracking tasks. However, this approach can lead to a reduction in tracking speed. In SiamHYPER [42], a spatial spectral cross-attention module was designed for the fusion of spectral features. However, this module only works on one branch. To obtain cross-band information from HSI, CBFF-Net [15] introduced a cross-band group attention (CBGA) module. However, the lack of spatial attention in this approach could lead to an inability to capture essential details within specific regions. Inspired by shuffle attention networks (SANet) [43], we designed the HOT method using the pyramid shuffle attention module, with the aim of improving similarity features to address challenges related to target scale variations and interference from similar objects.

### 2.3. Knowledge Distillation

Knowledge distillation has been extensively used for model compression and transfer learning. Its primary objective is to impart the knowledge from a complex model (teacher model) to a simplified model (student model). Hinton et al. [44] presented a groundbreaking KD design, which uses the output of the teacher network to help train the student network. Tarvainen et al. [45] and Liu et al. [46] proposed strategies for distilling knowledge from multiple teacher models to improve the efficiency of KD. Lin et al. [47] introduced a comprehensive framework that utilizes low-rank decomposition to eliminate redundancy between fully connected layers and convolutional layers. Yim et al. [48] adopted an information measurement-based approach to improve the efficiency of knowledge transfer. Sepahv et al. [49] decomposed the characteristic of their teacher model to obtain a core tensor, allowing the student to better comprehend the knowledge.

In summary, KD has been extensively applied in the field of DL, including in image classification [50,51,52], object detection [53,54,55], and semantic segmentation [56,57,58]. However, there is limited research that investigates the implementation of KD within the realm of hyperspectral target tracking.

## 3. Methodology

In this section, the proposed HOT is described in detail, including the Siamese model, the PSA model, and the KD of the hyperspectral data.

### 3.1. The SiamCAR Tracker

Crucial to the successful implementation of DL models is the ability to perform offline learning on massive datasets, enabling them to learn complex and intricate relationships from extensively annotated data. Generally, Siamese networks consider tracking a similarity learning task, which utilizes end-to-end offline training to comprehend the similarity between target images and search regions. Typically, tracking methods based on Siamese networks consist of two stages: feature extraction and bounding-box prediction. Initially, the template *Z* and the search region *X* are fed into two separate branches sharing the same parameter of the backbone network. It is crucial to fuse visual attributes (represented by low-level features) with semantic information referring to high-level features, which helps to accurately recognize the target. Specifically, the fused feature $\varphi \left(X\right)\in R^{7\times 7\times 768}$ of the search region concatenates the output of the final three residual blocks of the model (ResNet-50) [3], which is represented by

(1) $$\varphi \left(X\right)=C(F_{3}\left(X\right),F_{4}\left(X\right),F_{5}\left(X\right))$$

where C(·) denotes the concatenation function and $F_{3}\left(X\right)$, $F_{4}\left(X\right)$, and $F_{5}\left(X\right)$ represent the outputs of the final three blocks in backbone network. In addition, the fused feature φ(Z), which refers to the target template, is obtained similarly.

In order to incorporate information from the template and search branches, a deep cross-correlation is applied to φ(X) and φ(Z) to obtain a response feature map *M* with 256 channels.

(2) M=φ(X)★φ(Z)

where ★ denotes channel-wise correlation operations. *M* retains abundant information, which also increases the difficulty of prediction at a later stage.

The next stage of the prediction of the target box focuses on the task of classification and regression with extracted features. Another branch predicts the bounding box for the target. The classification branch outputs feature maps of size 25×25 with 2 channels. Each point on the classification feature map has a 2-D vector. This vector represents the scores for the target and background. The classification loss $L_{cls}$ utilizes the cross-entropy loss, the formula for which is as follows:

(3) $$L_{cls}=\frac{1}{N}\sum_{d}g_{d}\log\left(p_{d}\right)$$

where *N* represents the total number of points, $g_{d}$ is the ground-truth label for the d-th point, and $p_{d}$ represents the predictive probability for the d-th point.

The search branch outputs feature maps of size 25×25 with 4 channels. As a result, each point (i,j) on the regression feature map has a four-dimensional vector v(i,j)=(l,t,r,b), where *l* denotes the distance between a point within the search area and the left boundary of the real target. Similarly, *t*, *r*, and *b* refer to the upper boundary, the right boundary, and the lower boundary, respectively.

The center-ness branch assigns lower scores to points farther away from the target center, concentrating the predicted boxes around the target. This branch outputs a 25×25×1 feature map, where each value at a point (i,j) on the map corresponds to the centrality score of the corresponding position on the search map. The centrality score $S_{\mathrm{Cen}}\left(i,j\right)$ at the corresponding position in the predicted feature map is computed by

(4) $$S_{\mathrm{Cen}}\left(i,j\right)=Q\times \sqrt{\frac{\min(l,r)}{\max(l,r)}\times \frac{\min(t,b)}{\max(t,b)}}$$

where *Q* takes values of 0 or 1. If a point (i,j) is not within the manually marked bounding box of initial frame, *Q* is 0.

The loss function $L_{\mathrm{reg}}$ referring to the regression branch is calculated using the intersection over union (IOU) loss [59] $L_{\mathrm{IOU}}$ to regress the position of the predicted bounding box, as shown in Equation (5).

(5) $$L_{\mathrm{reg}}=\frac{1}{\sum Q}\sum_{i,j}Q\times L_{\mathrm{IOU}}\left(u\left(x,y\right),v\left(i,j\right)\right)$$

where u(x,y) represents the distance from the real target position (x,y) to the four sides of the ground-truth bounding box.

The loss function for the center-ness branch $L_{cen}$ is represented by

(6) $$\begin{array}{c} L_{\mathrm{cen}}=\frac{-1}{\sum Q}\sum (S_{\mathrm{Cen}}\left(i,j\right)\times \log D_{\mathrm{Cen}}\left(i,j\right)\\ +\left(1-S_{\mathrm{Cen}}\left(i,j\right)\right)\times \log\left(1-D_{\mathrm{Cen}}\left(i,j\right)\right)) \end{array}$$

where $D_{\mathrm{Cen}}\left(i,j\right)$ denotes the value at the corresponding position in the center-ness branch map.

Therefore, the final loss *L* is obtained by

(7) $$L=L_{\mathrm{cls}}+\lambda_{1}L_{\mathrm{cen}}+\lambda_{2}L_{\mathrm{reg}}$$

where $\lambda_{1}$ and $\lambda_{2}$ are penalty factors.

### 3.2. The Pyramid Shuffle Attention Module

**Figure 4:**
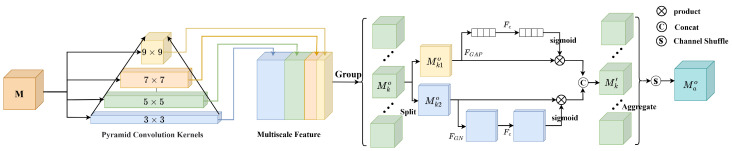
The PSA module. *M* represents the initial similarity features. Multi-scale features are obtained by pyramid convolution. These are further divided into multiple sub-features along the channel dimension. The dependencies between pixels and channels are captured through the parallel use of spatial and channel attentions.

Significant differences between color and HSIs make it difficult for most trackers to exploit the abundant spectral information. HSIs increase the complexity of similarity features in Siamese trackers, which in turn limits the performance of prediction modules. This limitation is particularly evident when dealing with scale variations, resulting in poor tracker performance. To address these issues, we introduce a pyramid shuffle attention module for similarity matching, the specific design of which is depicted in Figure 4.

Firstly, multiscale similarity features extracted through pyramid convolution [60] are grouped together. Next, the shuffle unit [43] is used to incorporate spatial and channel attention into a block. Finally, all sub-features are combined, benefiting from the channel shuffle.

**Figure 5:**
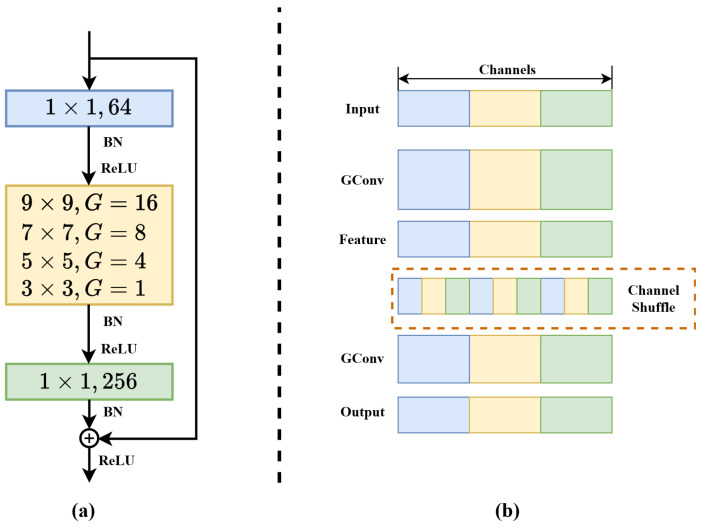
(**a**) Pyramid convolution. BN stands for Batch Normalization, and ReLU stands for Rectified Linear Unit. (**b**) Channel shuffle. The operation handles intergroup information exchange by dividing each group into several smaller blocks and recombining those blocks between the different groups.

A set of convolution kernels $K_{I=1,2,3,4}$ with different sizes, 3×3, 5×5, 7×7, and 9×9, is utilized to obtain fused features of multiple scales, as seen in Figure 5, which can be described as

(8) $$M_{I}^{o}=F_{\mathrm{gc}}\left(M,K_{I},G_{I}\right)$$

where $G_{I}$ represents the number of groups in the grouped convolution (GConv) $F_{\mathrm{gc}}\left(\cdot \right)$, which can control the connectivity in the convolution operation. Subsequently, the response feature maps $M_{I}^{o}$ of each pyramid level are concatenated along the channel dimension.

(9) $$M^{o}=C\left(M_{1}^{o},M_{2}^{o},M_{3}^{o},M_{4}^{o}\right)$$

where the fused feature map $M^{o}\in R^{V\times H\times W}$, *V*, *H*, and *W*, respectively, represent the number of channels and the spatial height and width. Then, $M^{o}$ is divided into *J* groups along the channel dimension, that is, $M^{o}=\left[M_{1}^{o},\cdots ,M_{J}^{o}\right]$. The shape of $M_{k}^{o}$ becomes (V/J)×H×W. Next, $M_{k}^{o}$ splits into two branches along the channel dimension, namely $M_{k1}^{o},M_{k2}^{o}\in R^{(V/2J)\times H\times W}$. Therefore, the first branch uses channel attention, while the other branch uses spatial attention.

For the channel attention branch, we first apply global average pooling (GAP) $F_{\mathrm{GAP}}$ to $M_{k1}^{o}$, reducing the feature map’s size from [(V/2J),H,W] to [(V/2J),1,1]. This is performed to integrate information from each channel and transform it into a global channel importance weight.

(10) $$F_{\mathrm{GAP}}\left(M_{k1}^{o}\right)=\frac{1}{H\times W}\sum_{m=1}^{H}\sum_{n=1}^{W}M_{k1}^{o}\left(m,n\right)$$

where $F_{\mathrm{GAP}}\left(M_{k1}^{o}\right)$ represents channel-wise statistical data, indicating the average importance of each channel in $M_{k1}^{o}$.

The channel attention $M_{k1}^{\prime }$ is calculated by

(11) $$M_{k1}^{\prime }=\sigma \left(F_{c}\left(F_{\mathrm{GAP}}\left(M_{k1}^{o}\right)\right)\right)M_{k1}^{o}$$

where $F_{c}$ denotes the convolution operation and σ(·) is a non-linear activation function.

Channel attention is used to improve the associations between channels, while spatial attention serves to understand the importance of different spatial positions. The spatial information $M_{k2}^{\prime }$ is obtained based on the second branch $M_{k2}^{o}$ with group normalization [61] $F_{GN}$.

(12) $$M_{k2}^{\prime }=\sigma \left(F_{c}\left(F_{\mathrm{GN}}\left(M_{k2}^{o}\right)\right)\right)M_{k2}^{o}$$

In the end, the output of the channel and the spatial attention branches is connected, that is, $M_{k}^{\prime }=\left[M_{k1}^{\prime },M_{k2}^{\prime }\right]\in R^{(V/J)\times H\times W}$. We aggregate all the sub-features $M_{k}^{\prime }$ and then perform channel shuffling [62] on the aggregated features, which is represented by

(13) $$M_{a}^{o}=S\left(C\left(M_{1}^{\prime },M_{2}^{\prime },\cdots ,M_{J}^{\prime }\right)\right)$$

where S(·) denotes the channel shuffling Figure 5, which enhances the information exchange between channels in the aggregated feature. $M_{a}^{o}$ is the final similarity feature, which provides a more powerful representation of the features for subsequent prediction of the target boxes.

### 3.3. Hyperspectral Knowledge Distillation

**Figure 6:**
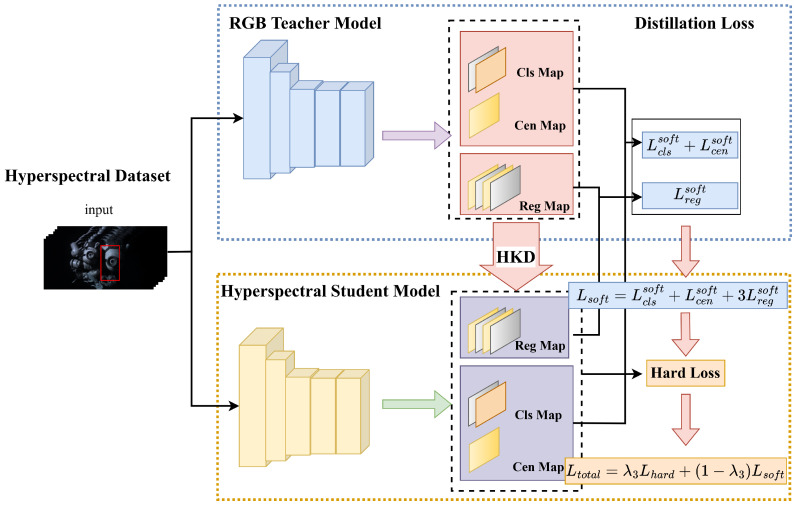
Hyperspectral knowledge distillation.

Due to the insufficient training data in the field of hyperspectral imaging, the overfitting problem easily arises when training DL-based trackers. To address these challenges, we designed a novel hyperspectral knowledge distillation approach aimed at transferring knowledge from an RGB model to a hyperspectral model. The objective was to improve the performance and generalizability of the hyperspectral model in situations where data availability is limited. HKD is a special knowledge distillation technique in which the teacher and student models have the same network architecture. As shown in Figure 6, we provide a tracking model that is specified as a teacher model trained on a comprehensive RGB dataset.

The student model designed in our study is an HOT model. In the realm of HKD, the process of training the student model is directed by the teacher model through the utilization of activation values derived from its output layer. In this study, we consider the final output of the prediction head in the tracking model to be logits encompassing both the classification and regression branches. Therefore, the output of the teacher model is regarded as soft targets, and the student model is trained by minimizing the discrepancy between its own output and that of the teacher model.

In the classification branch, we consider the classification results of the RGB tracking model as soft labels, which are used to instruct the training of the HOT model. Furthermore, the introduction of a temperature parameter *T* serves to improve the classification results, allowing them to encompass a greater amount of information. Here, the soft classification loss function $L_{\mathrm{cls}}^{\mathrm{soft}}$ can be calculated according to the Kullback–Leibler divergence [63].

(14) $$\begin{array}{c} L_{\mathrm{cls}}^{\mathrm{soft}}=T^{2}\times \sum \left(SM\left(A_{t}^{\mathrm{cls}}/T\right)\log\left(\frac{SM(A_{t}^{\mathrm{cls}}/T)}{SM(A_{s}^{\mathrm{cls}}/T)}\right)\right) \end{array}$$

where SM denotes the softmax function and $A_{s}^{\mathrm{cls}}$ and $A_{t}^{\mathrm{cls}}$ are classification branching results referring to the student and teacher models.

Subsequently, the soft regression loss is obtained by

(15) $$L_{\mathrm{reg}}^{\mathrm{soft}}=\frac{1}{\sum Q}\sum_{i,j}Q\times L_{\mathrm{IOU}}\left(u\left(x,y\right),v^{\mathrm{soft}}\left(i,j\right)\right)$$

where $v^{\mathrm{soft}}\left(i,j\right)$ represents the distances between the feature map position (i,j) and the four edges of the soft target.

Similarly, the calculation of centrality loss is represented by

(16) $$\begin{array}{c} L_{\mathrm{cen}}^{\mathrm{soft}}=\frac{-1}{\sum Q}\sum (S_{\mathrm{Cen}}^{\mathrm{soft}}\left(i,j\right)\times \log D_{\mathrm{Cen}}\left(i,j\right)\\ +\left(1-S_{\mathrm{Cen}}^{\mathrm{soft}}\left(i,j\right)\right)\times \log\left(1-D_{\mathrm{Cen}}\left(i,j\right)\right)) \end{array}$$

where $S_{\mathrm{Cen}}^{\mathrm{soft}}\left(i,j\right)$ represents the centrality score calculated using the soft target.

Therefore, the total loss calculation is formulated as

(17) $$L_{\mathrm{total}}=\lambda_{3}L+\lambda_{4}\left(L_{\mathrm{cls}}^{\mathrm{soft}}+L_{\mathrm{cen}}^{\mathrm{soft}}+3L_{\mathrm{reg}}^{\mathrm{soft}}\right)$$

where the hard loss *L* is calculated by Equation (7). Furthermore, $\lambda_{3}$ and $\lambda_{4}$ denote penalty factors.

**Figure 7:**
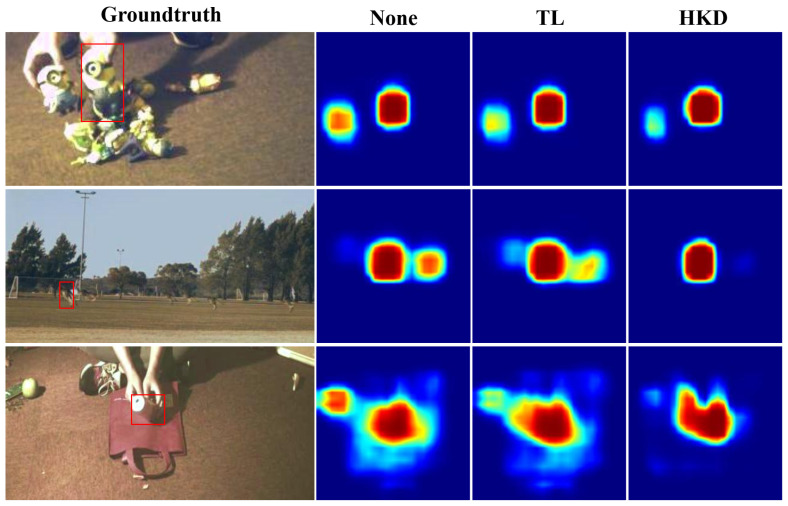
Visualization of response maps in three videos (from top to bottom, toy1, kangaroo, and coke). The red box indicates the ground truth. Specifically, ‘None’, ‘TL’, and ‘HKD’ represent the baseline tracker, the baseline tracker with TL, and the baseline tracker with HKD, respectively.

To prove the performance of HKD, we visualize the response maps under three different strategies in the toy1, kangaroo, and coke videos in Figure 7. In the response map of the baseline algorithm, similar objects also exhibit high response values, which may lead to deviations in the tracker. When training the tracker using the TL and HKD methods, a significant reduction in response to distracting objects was observed, especially with the HKD training method, where the effect was more pronounced.

## 4. Experiments

### 4.1. Experimental Data

The GOT10K [19] dataset was taken as the training set for the teacher model in this article. The dataset consists of over 1000 unique color video sequences, totaling more than 180,000 frames. For the training of the HOT model, the HOT2022 [5] dataset was used. This was provided by the Hyperspectral Target Tracking 2022 (HOT2022) Challenge, located at hsitracking.com, (accessed on 28 November 2023). The dataset consists of 40 training sequences and 35 test sequences, with a mean size of 500 frames per sequence. It encompasses a diverse range of objects, scenes, and activities. Additionally, each video is annotated with 11 challenging attributes [20], covering factors such as occlusion (OCC), out-of-view (OV), background clutter (BC), deformation (DEF), motion blur (MB), fast motion (FM), low resolution (LR), out-of-plane rotation (OPR), illumination variation (IV), in-plane rotation (IPR), and scale variation (SV). These datasets provide us with rich, diverse, and challenging benchmarks that contribute to the advancement of HOT technology.

### 4.2. Experimental Setup

The proposed method is executed using PyTorch and runs on a GTX 3080 GPU. We set the input size of the template and search area to 127 pixels and 255 pixels, respectively. We used ResNet-50, as proposed in [18], which served as our Siamese subnetwork. This network was pre-trained on ImageNet [64] and employed its parameters for retraining within our model. In the teacher model, the batch size was set as 80, the epoch number was 20, and the initial learning rate (LR) was 0.001. During the initial 10 epochs, the training process involved keeping the parameters of the Siamese subnetwork fixed while focusing on training the classification and regression subnetworks. However, in the concluding 10 epochs, the concurrent training approach was adopted, which involved unfreezing the last three blocks of ResNet-50. The teacher model was trained with the GOT-10K dataset, while the HOT2022 hyperspectral dataset was utilized to train our tracker. Furthermore, the LR was then changed to 0.0005. *T* in Equation (14) was set to 2. In the loss function, $\lambda_{1}=1$, $\lambda_{2}=3$, $\lambda_{3}=0.9$, and $\lambda_{4}=0.1$. The testing details were identical to those in [6], employing an offline tracking strategy. Only the objects in the initial frame of the video were considered templates.

### 4.3. Experimental Results and Analysis

#### 4.3.1. Qualitative Comparison

We conducted a qualitative comparison between SOTA trackers, including DaSiamRPN [24], SiamRPN++ [23], DeepHKCF [7], BS-SiamRPN [65], ADSiamRPN [12], MHT [5], MFIHVT [9], and BAENet [11]. The first two are RGB trackers, while the rest are hyperspectral trackers. BAENet is a method that divides HSIs into multiple three-channel images by learning the nonlinear relationship between different spectral bands and generating band weights. It employs ensemble learning to fuse weak trackers, enabling end-to-end training and achieving excellent performance. MFIHVT [9] uses a pre-trained VGG-19 network and an oriented gradient histogram to extract convolutional features, which are merged to produce the ultimate feature representation. MFIHVT employs a kernelized correlation filter framework for target detection in hyperspectral videos and utilizes adaptive weight coefficients to fuse response maps generated from different features. Additionally, MFIHVT incorporates scale estimation and dimensionality reduction strategies to improve tracking robustness and efficiency. MHT [5] integrates spatial spectral information into multidimensional gradient histograms and global material features to construct hand-made features, which are combined with the BACF [66] framework to fuse material-based features and locate the target accurately. MHT effectively encodes the spatial spectral structure and material composition of HSIs using material information, thereby improving tracking performance. ADSiamRPN [12] uses a genetic optimization algorithm to select informative spectral bands. Additionally, it utilizes the SiamRPN model to extract hyperspectral features for target localization and classification. In addition, HSUpdateNet, a designed network, is introduced to obtain accumulated templates and address target deformation issues. BS-SiamRPN [65] utilizes intelligent optimization algorithms to determine key spectral bands. It employs TL to acquire semantic information and utilizes specific spectral band information as a network input for target matching. DeepHKCF [7] is an HOT based on deep kernel-based correlation filtering. It involves the transformation of HSIs into false-color images, followed by the extraction of deep features with a VGG-19 network. SiamRPN++ [23] is a Siamese network-based tracking algorithm that uses deep models to learn similarity maps. Deep network architectures and feature pyramid networks can enhance detection performance. The primary contribution of SiamRPN++ lies in its effective reduction in the impact of negative samples through the hard negative mining training strategy, improving the tracker’s performance. The DaSiamRPN method [24] improves the discriminative capacity of the Siamese network, introduces an interference-aware module to suppress the influence of distractors, and utilizes a local-to-global search strategy to achieve offline training and online tracking objectives.

**Figure 8:**
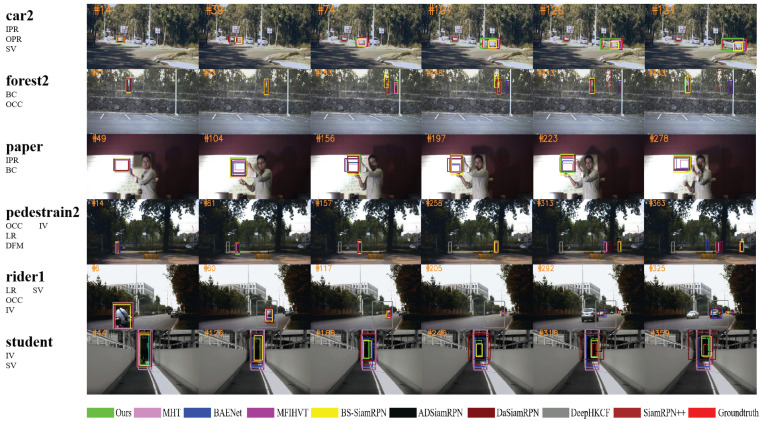
The qualitative comparison of our tracker and other trackers in several videos (from top to bottom, car2, forest2, paper, pedestrain2, rider1, and student).

Figure 8 presents the comparative result of these trackers in six challenging video sequences, namely car2, forest2, paper, pedestrian2, rider1, and student. The resolution and frame count of the six sequences are shown in Table 1. In the car2 video, a white car approaches from a distance, gradually increasing in size. Simultaneously, there are two similar cars parked on the roadside. Both SiamRPN++ and DeepHKCF fail to achieve effective discrimination and separation between targets and distractors. This finding demonstrates that hyperspectral features can help to effectively distinguish color-similar distractors. The DeepHKCF model is based on a single depth feature, resulting in the insufficient use of hyperspectral features. In frame #120, it is evident that the aspect ratio of the white car changes, causing all algorithms except our proposed one to fail in accurately enclosing the target. Our algorithm’s success is attributed to the PSA module, which fuses multi-scale features into the similarity map. This enables the tracker to more accurately capture target changes.

Within the forest2 video, the target subject wearing a green jacket navigates through a wooded area. For target occlusion, the absence of the utilization of spectral information in DaSiamRPN and SiamRPN++ results in the loss of target tracking. After frame #313, both BAENet and MFIHVT fail to maintain target tracking and are unable to reestablish tracking in their subsequent course of action.

In the paper video, a blank sheet of paper continuously flips and moves against a similar background. When the target undergoes frequent changes, most of the trackers do not respond adequately to these changes. However, our anchor-free tracker is not limited by specific anchor boxes. Therefore, it exhibits good robustness to target deformations, rotations, and other appearance variations, allowing accurate target tracking in complex scenarios.

In the pedestrian2 video, the pedestrian in front of the tree transitions from bright to dark. In the subsequent target motion, there are occurrences of tree occlusion, causing the target to temporarily disappear from the screen, which presents significant challenges for the trackers. During this tracking process, BAENet struggles to avoid interference from similar objects. When the target experiences occlusion, the HOTs (MFIHVT and MHT) also exhibit tracking migration, making it difficult to reestablish tracking in the following frames. Our method can track the target again even after the target is blocked. This is because the training method of HKD uses color data to learn general computer vision knowledge, such as context information and semantic information.

Within the rider1 video, the target encounters challenges of illumination variations, scale changes, and low resolution. The DeepHKCF tracker struggles to deal effectively with scale variation because it is reliant upon a sole depth feature. BAENet lost the target after 292 frames due to interference in the redundant bands, which caused the tracker to not be able to discriminate similar objects well enough. Due to the enhanced similarity features of the PSA module, our tracker can distinguish targets even in complex environments.

In the student video, a student target continues to move; its shape changes and gradually fades into darkness. All trackers were able to track the target, but after 188 frames, none of the trackers could fit the target except our tracker and BS-SiamRPN. In summary, the proposed tracker shows robustness in handling these challenges and achieves good tracking performance in this video.

#### 4.3.2. Quantitative Comparison

In this section, we used four comprehensive evaluation metrics, namely precision plots, success plots, average distance precision at a threshold of 20 pixels (DP@20P), and AUC. The success plot illustrates the percentage curve of overlap scores (OS) greater than a designated threshold, where the OS represents the IOU ratio between the predicted bounding box and the ground-truth bounding box. The precision plot is a percentage curve of video frames in which the distance between the predicted bounding box and the ground truth bounding box is less than a given threshold. Furthermore, DP@20P denotes the value of the precision plot when the threshold is set to 20. The AUC computes the area beneath the success plot curve, reflecting the tracker’s average performance across various overlap ratios. All results are obtained by one-pass evaluation (OPE), which initializes the tracker using the target ground truth from the first frame of the tracking sequence.

**Figure 9:**
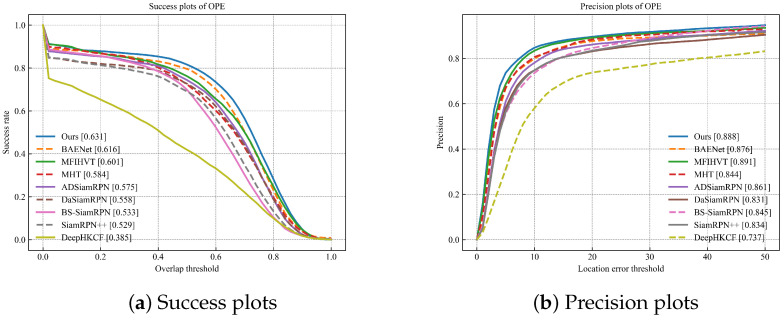
Success rate and precision rate referring to overall videos.

**Figure 10:**
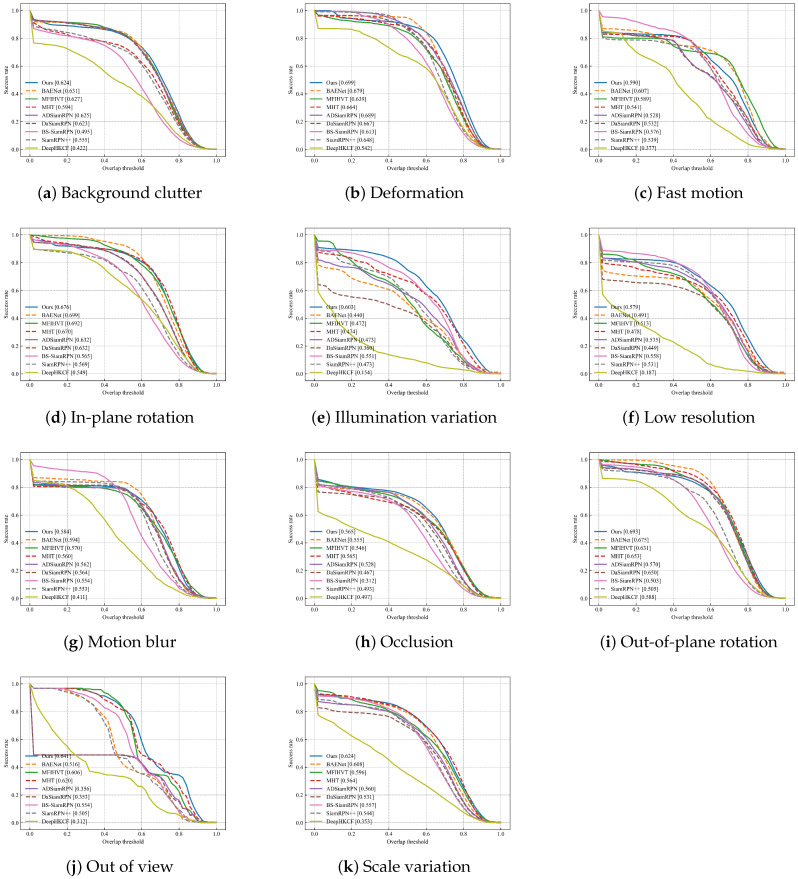
The success plot for the HOT2022 dataset for eleven challenges.

**Figure 11:**
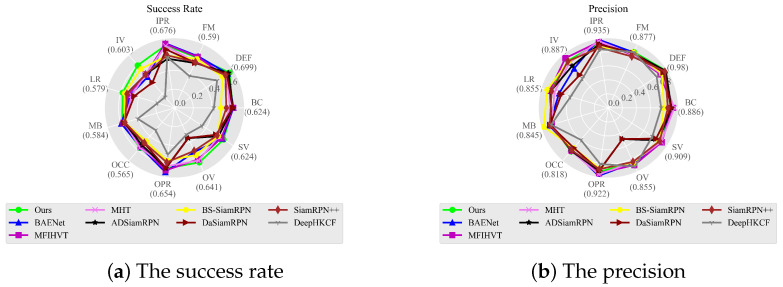
The success rate and precision of eleven challenges for the overall videos.

The success and precision plots of these trackers are shown in Figure 9. In Table 2, it can be observed that compared to the BAENet method, SiamPKHT achieves a 2.4% increase in the success rate and a 1.4% improvement in precision. Although the precision of MFIHVT is slightly higher than that of our method, its success rate is lower, with a value of 0.03. Moreover, this method suffers from a slow tracking speed and does not meet real-time requirements. To gain deeper insights into the performance of SiamPKHT, we evaluated 9 trackers among the 11 attributes of the sequences. Success plots of these trackers for the 11 distinct attributes are shown in Figure 10. In Table 3 and Table 4, it is observed that SiamPKHT performs the best in several attributes (DEF, IV, LR, OCC, OV, and SV) and second best in FM and MB when referring to both the success rate and the precision. This is due to the guidance of a large color dataset during the HKD process, which helps the model learn common features and capabilities. In Figure 11a, it can also be seen that SiamPKHT exhibits excellent performance in all challenges, particularly for LR and SV, which significantly outperforms other SOTA tracking algorithms. This is attributed to the pyramid shuffle attention module. This module enhances differences in similarity and captures changes in target appearance by fusing information from different scales.

#### 4.3.3. The Ablation Study

To assess the effectiveness of each part of the SiamPKHT tracker, an ablation study was performed on the HOT2022 benchmark. The five different testing models were as follows.

1. Baseline: The SiamCAR model trained solely on the GOT10K dataset was used as the baseline model.
2. Baseline-PSA: The baseline exclusively utilizing the PSA module.
3. Baseline-PSA-TL: The baseline with both the PSA module and transfer learning.
4. Baseline-PSA-SelfKD: The baseline with both the PSA module and self-knowledge distillation.
5. Baseline-PSA-HKD: The baseline with both the PSA module and hyspectral knowledge distillation.

In the baseline model, the tracker was first trained on the GOT10K dataset and then tested using false-color sequences from HOT2022. In the Baseline-PSA model, only the PSA module was utilized without employing TL with hyperspectral data. The Baseline-PSA-TL model fine-tuned the Baseline-PSA model using hyperspectral data and served as the student model in the Baseline-PSA-HKD model. The Baseline-PSA-SelfKD model incorporated hyperspectral data and applied self-knowledge distillation [67] for TL from the Baseline-PSA model. Finally, the Baseline-PSA-HKD model combined all the proposed tracker components.

**Figure 12:**
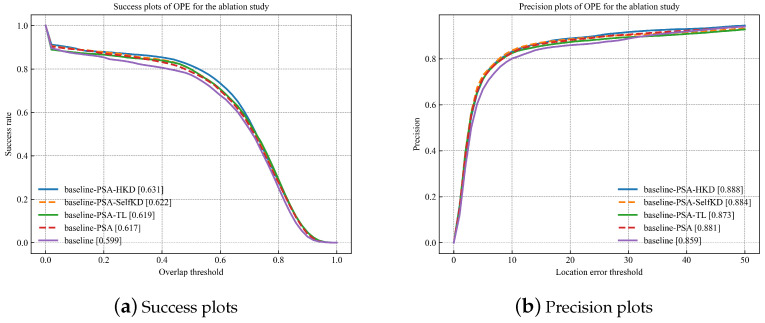
The ablation study with all test HSVS.

The evaluation curves and the effectiveness of each component referring to the proposed method are depicted in Figure 12. Compared to the first two models, models 3, 4, and 5 exhibit better AUC performance because these models take advantage of the hyperspectral data. In Table 5, with PSA, our method obtains a better result (+0.018) with respect to AUC scores. Furthermore, it can be seen that the integration of PSA and HKD obtains a notable increase (0.032) in the AUC score, improving overall performance. We attribute these benefits to the inclusion of more spectral information in HSIs compared to color images. The additional spectral information provides a richer feature representation, which helps the tracker better understand the nature of the observed objects. Due to the complexity of HSVs, traditional prediction and decoding methods may struggle to effectively extract accurate information from targets. The proposed PSA module explores feature information at different depths along the channel dimension, allowing the tracker to more fully utilize the abundant information in the HSVs during the subsequent prediction stage. In addition, the HKD module overcomes the limitation of limited hyperspectral data, which mitigates the risk of overfitting in transfer learning and effectively obtains valuable information from the hyperspectral data.

## 5. Conclusions and Future Work

In this article, we introduced the SiamPKHT method, which integrates pyramid shuffle attention (PSA) and knowledge distillation (HKD) into the SiamCAR framework for enhanced object tracking. The PSA module significantly improves the model’s perception capabilities, while the HKD addresses overfitting issues due to limited hyperspectral data, thereby improving the tracker’s generalizability. Our experimental results on HOT2022 demonstrate that SiamPKHT outperforms baseline trackers and shows substantial competitiveness against state-of-the-art (SOTA) HOT methods, achieving a remarkable tracking speed of 43 FPS, which is suitable for real-time applications.

However, the proposed SiamPKHT, despite its promising performance, encounters challenges in scenarios involving occlusion and low-resolution environments. These limitations highlight the need for further research in these areas. Future work will also focus on refining feature extraction techniques for hyperspectral videos (HSVs), aiming to mitigate the limitations posed by insufficient visible data. This exploration is crucial for advancing the field and ensuring that our method remains relevant and effective in a broader range of tracking scenarios. 

## Figures and Tables

**Table 1:** The details of the experimental videos.

Video	Car2	Forest2	Paper	Pedestrian2	Rider1	Student
Frame	131	363	278	363	336	396
Resolution	351 × 167	512 × 256	446 × 224	512 × 256	512 × 256	438 × 256

**Table 2:** Performance comparison with other SOTA trackers (Red and brown fonts denote the best and sub-optimal results, respectively).

Algorithm	Video Type	AUC	DP@20P	FPS
DaSiamRPN	false-color	0.558	0.831	48
SiamRPN++	false-color	0.529	0.834	41
DeepHKCF	HSV	0.385	0.737	2
BS-SiamRPN	HSV	0.533	0.845	55
ADSiamRPN	HSV	0.575	0.861	35
MHT	HSV	0.584	0.876	2
MFIHVT	HSV	0.601	0.891	2
BAENet	HSV	0.616	0.876	≈1
Ours	HSV	0.631	0.888	43

**Table 3:** Attribute-based comparisons of the success rate (Red and brown fonts denote the best and sub-optimal results, respectively).

Attributes	Ours	BAENet	MFIHVT	MHT	ADSiamRPN	BSSiamRPN	DeepHKCF	SiamRPN++	DaSiamRPN
BC	0.624	0.631	0.627	0.594	0.625	0.495	0.422	0.555	0.623
DEF	0.699	0.679	0.639	0.664	0.689	0.613	0.542	0.648	0.667
FM	0.590	0.607	0.589	0.541	0.528	0.576	0.377	0.539	0.532
IPR	0.676	0.699	0.692	0.670	0.632	0.565	0.549	0.569	0.632
IV	0.603	0.440	0.472	0.474	0.473	0.551	0.154	0.473	0.360
LR	0.579	0.491	0.513	0.478	0.535	0.558	0.187	0.531	0.449
MB	0.584	0.594	0.570	0.560	0.562	0.554	0.411	0.553	0.564
OCC	0.565	0.555	0.546	0.565	0.528	0.467	0.312	0.493	0.497
OPR	0.654	0.693	0.675	0.631	0.653	0.570	0.503	0.588	0.650
OV	0.641	0.516	0.606	0.620	0.356	0.554	0.312	0.505	0.353
SV	0.624	0.608	0.596	0.564	0.560	0.557	0.353	0.544	0.531

**Table 4:** Attribute-based comparisons of the precision (Red and brown fonts denote the best and sub-optimal results, respectively).

Attributes	Ours	BAENet	MFIHVT	MHT	ADSiamRPN	BSSiamRPN	DeepHKCF	SiamRPN++	DaSiamRPN
BC	0.886	0.908	0.918	0.942	0.901	0.795	0.755	0.85	0.899
DEF	0.98	0.94	0.885	0.901	0.975	0.908	0.82	0.963	0.937
FM	0.877	0.871	0.832	0.841	0.859	0.872	0.874	0.807	0.859
IPR	0.935	0.985	0.951	0.964	0.918	0.873	0.846	0.868	0.914
IV	0.887	0.745	0.939	0.85	0.791	0.876	0.56	0.881	0.621
LR	0.855	0.733	0.872	0.801	0.853	0.912	0.563	0.846	0.699
MB	0.845	0.881	0.841	0.844	0.855	0.96	0.851	0.869	0.867
OCC	0.818	0.79	0.811	0.812	0.81	0.756	0.601	0.801	0.754
OPR	0.922	0.978	0.942	0.958	0.906	0.875	0.809	0.883	0.898
OV	0.855	0.864	0.895	0.851	0.488	0.859	0.918	0.846	0.488
SV	0.909	0.907	0.905	0.895	0.856	0.864	0.748	0.873	0.811

**Table 5:** The ablation study with all test HSVs (Red fonts denote the best results).

Algorithm	Video Type	AUC	DP@20P
Baseline	false-color	0.599	0.859
Baseline-PSA	HSV	0.617	0.881
Baseline-PSA-TL	HSV	0.619	0.873
Baseline-PSA-SelfKD	HSV	0.622	0.884
Baseline-PSA-HKD	HSV	0.631	0.888

## Data Availability

Data are available from the corresponding author upon reasonable request.
